# Rare Case of Pulmonary Perivascular Epithelioid Cell Tumor (PEComa) Causing Right Lung Collapse Managed With Bronchial Stenting: A Clinical Challenge

**DOI:** 10.7759/cureus.94753

**Published:** 2025-10-16

**Authors:** Milan N Regmi, Mohamad N Jajeh, Roger Lin, Kevin Meek, Mina Saba

**Affiliations:** 1 Internal Medicine, Southeast Health Medical Center, Dothan, USA

**Keywords:** case report, endobronchial stenting, palliative treatment, pecomas, radiological finding

## Abstract

Pulmonary perivascular epithelioid cell tumor (PEComa) is an extremely uncommon mesenchymal tumor; pulmonary PEComas can present serious difficulties for both diagnosis and treatment. We present the case of a 62-year-old male with a history of unresectable PEComa of the upper back with metastatic progression to the lungs. Despite ongoing sirolimus therapy, the patient experienced significant tumor growth, presenting to the emergency department with respiratory distress, and was found to have a rapidly enlarging pulmonary mass, later referred for bronchial stenting for palliative treatment. Due to the rarity of these tumors, diagnosis often requires a high index of suspicion and relies on histopathologic confirmation, including immunohistochemistry for melanocytic and muscle markers. Imaging alone is insufficient to distinguish PEComas from other pulmonary masses, underscoring the need for tissue diagnosis. Furthermore, the clinical behaviour of pulmonary PEComas is unpredictable; some remain indolent, while others exhibit aggressive metastatic potential. This heterogeneity complicates treatment planning and highlights the need for individualized, multidisciplinary approaches. We highlight the fact that pulmonary PEComa can rapidly progress and can cause significant morbidity. Palliation with bronchial stenting can be a reasonable option.

## Introduction

Perivascular epithelioid cell tumors (PEComas) are a family of mesenchymal tumors characterized by a unique cell type called the perivascular epithelioid cell (PEC), which is more common in females than males, with a rare incidence of around 0.3 per million population. Approximately 42 to 84 new diagnoses of PEComa occur in the USA every year [1,2]. PEComas are epithelioid or spindle cells that exhibit a unique immunohistochemical stain pattern, similar to that of smooth muscle cells and melanocytic cells, and are positive for HMB-45 and/or Melan-A, as well as smooth muscle markers such as actin and desmin [2]. 

We are presenting a case of PEComa of the lungs, how it progresses to the advanced stage, and treatment in critical care settings.

## Case presentation

This is a case of a 62-year-old male with history of a painless lump on his back that was progressively enlarging since one year, at the time of initial assessment one year back, a mediastinal mass of 7.7 x 7.2 x 7.4 cm was discovered along with mass in his back measuring 4.7 x 5.5 x 6.2 cm; a biopsy was done, revealing malignant epithelioid neoplasia favoring PEComa. Immunohistochemistry was positive for HMB45, E-cadherin, desmin, and cathepsin. As the mass kept on growing, he started to have severe pain and restlessness. On further assessment of imaging, the mass grew to such a size that it was not resectable. He was started on oral sirolimus and radiation therapy to help decrease the bulk and his symptoms. Pain was adequately controlled.

As he was getting treatment, his tumor progressed over a period of one year to his cervical and thoracic spine with spinal cord compression, for which neurosurgery was consulted. Given the vascularity of the tumor, its aggressive nature, and the likelihood of recurrence, it was decided that neurosurgical intervention at this time would not lead to an improved outcome and carry far more risk than benefit, so they deferred surgery. Also, radiation oncology was consulted, but they deferred radiation as they felt no role for radiation therapy at this time, considering the close proximity of the previous site of radiation therapy and the risk of radiation-induced myelopathy.

**Figure 1:**
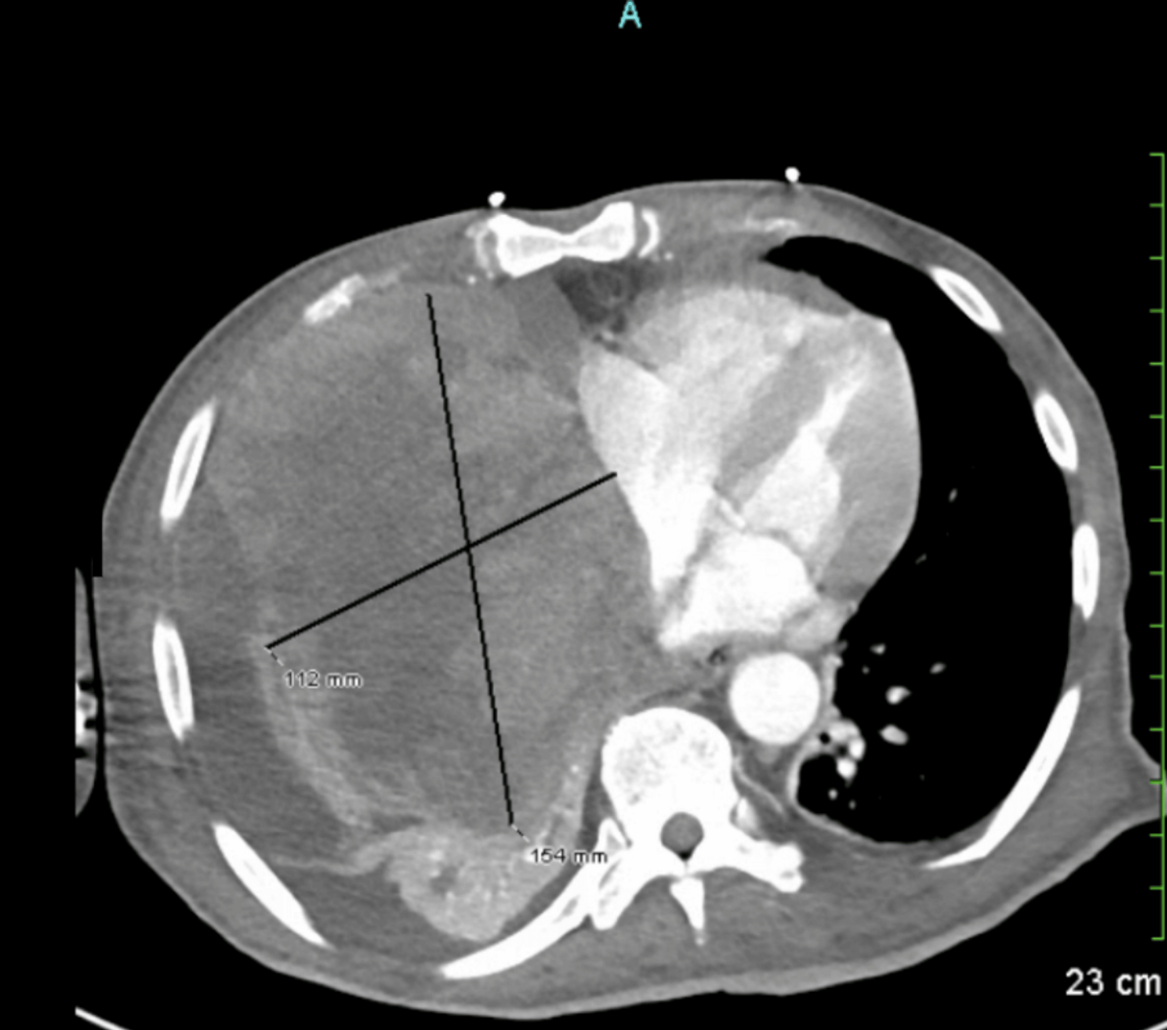
CT scan of the chest showing a large heterogeneous soft tissue tumor within the right thoracic cavity, invading the mediastinum and measuring 11 × 15 × 16 cm. Associated findings include a moderate right malignant pleural effusion, near-total collapse of the right lung, compression and collapse of the right heart chambers, and numerous pulmonary metastases. Numerous osseous metastases are also noted in the cervical spine.

**Figure 2:**
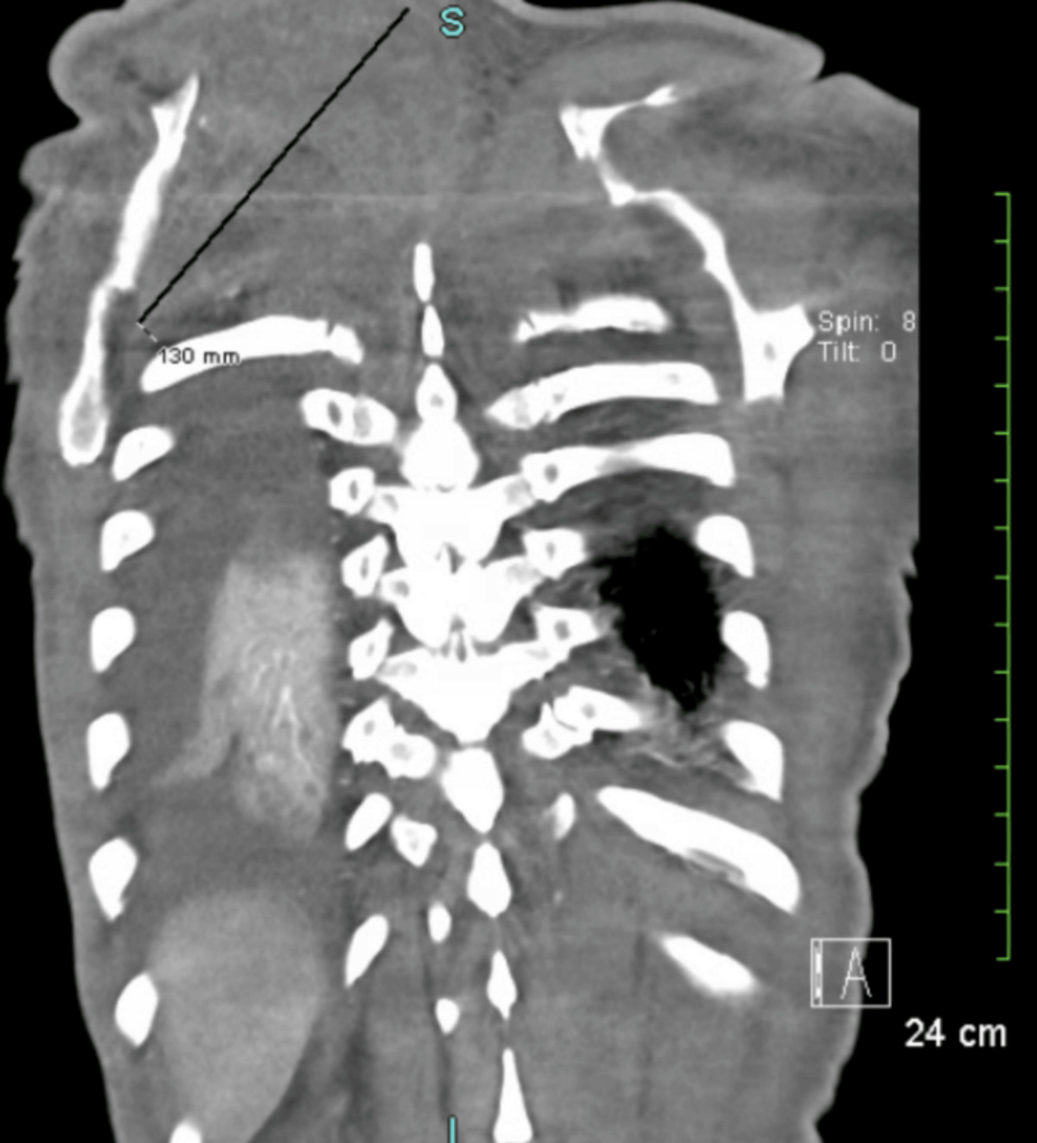
Large soft tissue tumor within the right dorsal back, measuring 14 × 7 × 13 cm, causing displacement of the scapula.

After one year of tumor diagnosis, the patient developed acute onset severe shortness of breath; Emergency Medical Service (EMS) was called, and upon EMS arrival, the patient's SpO_2_ was found to be at 62% breathing in ambient air, following which the patient was transported to the emergency room, where he was placed on oxygen through a nasal cannula at a rate of 5 L per minute, with oxygen saturation around 92%. CT of the chest, abdomen, and pelvis with IV contrast was performed and revealed a large, heterogeneous soft tissue tumor within the right intrathoracic chest invading the mediastinum, measuring 11 x 15 x 16 cm, associated with moderate right malignant pleural effusions with near total right lung collapse and collapse of the right heart chambers with numerous osseous metastases to the cervical spine; a large soft tissue tumor within the right dorsal back causing displacement of the scapula, diffuse anasarca, and ascites were also noted (Figures 1-2). Considering the total right lung collapse and the patient's hypoxemia, the patient was intubated at the ED, and plans for bronchoscopy were made. After intubation, the patient was noted to be hypotensive and started on Levophed and transferred to the ICU for further monitoring and evaluation. A transthoracic echocardiogram was done, revealing a right atrial mass by extrinsic, and trace pericardial effusion of no hemodynamic significance.

In one year, the size of the tumor growth in the anterior mediastinal mass has shown rapid growth and has doubled in size, increasing from 7.7 x 7.2 x 7.4 cm to 11 x 15 x 16 cm, which has caused significant atelectasis of the right middle and right lower lung lobes. To open up the lungs, he underwent bronchoscopy due to persistent lung collapse and was referred to a tertiary centre for bronchial stenting and opening up the collapsed lungs to help with oxygenation.

## Discussion

PEComas are under group of highly diverse group of soft tissue malignancies, composed of PECs, and are labelled as ultra-rare tumors with an estimated incidence of 0.3 per million [1]. Due to the uncertain malignant potential of PEComas, Folpe et al. [3] proposed a classification system that categorizes these tumors into three risk groups: benign, of uncertain malignant potential, and malignant. Although most PEComas are considered benign, four out of five tumors are diagnosed when they cause symptoms while compressing an adjacent organ. They are related to a tumor family that can arise in any part of the body, including myo-melanocytic immunophenotype [4]. The mainstay treatment for PEComas consists of radical resection for select patient given their resistance to standard chemotherapy and radiotherapy. For the treatment of unresectable tumors, mammalian target of rapamycin (mTOR) inhibitors and vascular endothelial growth factor receptor (VEGFR) inhibitors are used. Generally, PEComa are benign but often can be locally aggressive and malignant, causing bronchial stenosis and spinal invasion, which can cause severe morbidity, including intractable pain and collapse of lungs due to overgrowth of the mass locally. When the lungs are totally collapsed, stenting of the bronchus can be an option that can be palliative or bridging to surgical correction.

Tracheobronchial stenting has a long history and was used for the first time in 1952 when Harkins relieved tracheal stenosis with a metal tube [5]. Commonly, bronchial stents are used to relieve the obstruction and help with symptoms of patients, often caused by airway obstruction, as more than 80% of lung cancer patients are not eligible for curative surgical treatment; palliative relief of obstruction can be achieved by bronchial stenting, as in our patient who underwent palliative stenting [6].

The long-term outcome of stenting depends on the pathophysiology, severity, and nature of the underlying disease; for benign disease, stenting of the bronchus has a good outcome, whereas in non-resectable tumors or aggressive cancer, it is a palliative measure [7].

## Conclusions

Mesenchymal tumors that are extremely uncommon, pulmonary PEComas, can present serious difficulties for both diagnosis and treatment. For localized disease, surgical resection is the recommended course of treatment; however, systemic therapy with mTOR inhibitors is frequently necessary for cases that are metastatic or unresectable. Yet, as our patient showed, tumor growth in spite of medication treatment may call for palliative measures like bronchial stenting in order to reduce airway blockage and enhance respiratory function. More research is required to determine the best management strategies for pulmonary PEComas because there are currently no standardized treatment protocols.
